# Exploring *Trypanosoma cruzi* transmission dynamics in an acute Chagas disease outbreak using next-generation sequencing

**DOI:** 10.1186/s13071-024-06445-9

**Published:** 2024-09-18

**Authors:** Lissa Cruz-Saavedra, Carlos Ospina, Stivenn A. Gutiérrez, Jeiczon Jaimes-Dueñez, Omar Cantillo-Barraza, Carolina Hernández, Francisco Álvarez, María Blanco, Bernardo Leal, Lida Martínez, Manuel Medina, Mabel Medina, Silvia Valdivieso, Lauren Natalia Ramirez Celis, Luz H. Patiño, Juan David Ramírez

**Affiliations:** 1 Centro de Investigaciones en Microbiología y Biotecnología-UR (CIMBIUR), Facultad de Ciencias Naturales, Universidad del Rosario, Bogotá, Colombia; 2 Grupo de Investigación en Ciencias Animales-GRICA, Facultad de Medicina Veterinaria y Zootecnia, Universidad Cooperativa de Colombia (UCC), Bucaramanga, Colombia; 3 Grupo BCEI, Universidad de Antioquia, Medellín, Colombia; 4 Centro de Tecnología en Salud (CETESA), Innovaseq SAS, Bogotá, Colombia; 5 Programa de Control de ETV, Secretaría de Salud de Boyacá, Tunja, Colombia; 6 Secretaría Departamental de Salud de Arauca, Arauca, Colombia; 7 Grupo de Vigilancia en Salud Pública, Secretaría de Salud de Boyacá, Tunja, Colombia; 8 Hospital del Sarare Empresa Social del Estado, Saravena, Arauca, Colombia; 9 Molecular Microbiology Laboratory, Department of Pathology, Molecular and Cell-Based Medicine, Icahn School of Medicine at Mount Sinai, New York, NY USA

**Keywords:** Oral Chagas outbreak, *Trypanosoma cruzi*, *Trypanosoma rangeli*, DTU, Long-amplicon-based sequencing, Coinfection, Mixed infection, Loss of diversity

## Abstract

**Background:**

Chagas disease (CD), caused by *Trypanosoma cruzi*, poses a major global public health challenge. Although vector-borne transmission is the primary mode of infection, oral transmission is increasingly concerning.

**Methods:**

This study utilized long-amplicon-based sequencing (long-ABS), focusing on the 18S rRNA gene, to explore *T. cruzi*’s genetic diversity and transmission dynamics during an acute CD outbreak in Colombia, an area without domestic infestation.

**Results:**

Analyzing samples from five patients and five *T. cruzi*-positive marsupial samples, we identified coinfections between *T. cruzi* and *Trypanosoma rangeli*, mixed *T. cruzi* DTUs, suggesting possible links between human and marsupial *T. cruzi* infections. Coexistence of TcI, TcIV and *T. rangeli* suggests marsupial secretions as the possible source of *T. cruzi* transmission. Our investigation revealed diversity loss in DTUs TcIV and *T. rangeli* in humans after infection and in marsupial samples after culture.

**Conclusion:**

These findings provide significant insights into *T. cruzi* dynamics, crucial for implementing control and prevention strategies.

**Graphical abstract:**

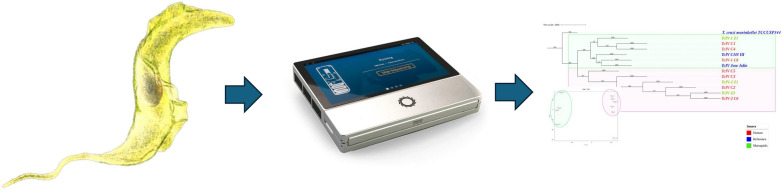

**Supplementary Information:**

The online version contains supplementary material available at 10.1186/s13071-024-06445-9.

## Background

Chagas disease, caused by the flagellate protozoan *Trypanosoma cruzi*, presents a grave global health challenge. The current estimates underscore the magnitude of the issue, with over 10 million people worldwide infected with the parasite. Even more alarmingly, over 100 million individuals are exposed to the risk of infection [1].

Chagas disease transmission primarily involves infected triatomine insects (vectorial transmission), but oral transmission is becoming a growing concern. This mode of transmission occurs when individuals consume contaminated food or water containing infective forms of *T. cruzi* from infected triatomine insects or marsupials [2]. Oral transmission has gained significant attention due to increasing cases reported in countries like Colombia, Venezuela and others [3–5]. A 2007 outbreak in Caracas, which infected 106 individuals, was one of the first indications of oral microepidemics [5]. These outbreaks lead to severe symptoms, with an average mortality rate reaching 6.51% [6], often concentrated in rural areas with *T. cruzi* reservoirs and vectors. This underscores the urgency of addressing oral transmission in efforts to control emergent forms of Chagas disease in endemic regions.

*Trypanosoma cruzi* exhibits remarkable genetic diversity, categorized into six Discrete Typing Units (DTUs, TcI-TcVI) and one genotype (TcBat) [7]. Intriguingly, even within the same DTU, variations can be observed [8, 9]. These distinct DTUs correlate with different host species, vectors, clinical manifestations, tissue preferences, treatment responses and disease severities [7]. The intricacy of *T. cruzi* infection is further highlighted by coinfections with other trypanosomatids and mixed infections involving various DTUs, illustrating the complex nature of the disease [10, 11]. Importantly, while the DTU TcI is primarily associated with most documented oral outbreaks, other DTUs like TcII, TcIII, TcIV and TcVI have also been linked [4, 12–14]. The methodologies based on molecular gene amplification or MLST approaches employed to study and surveillance these oral outbreaks have lacked the comprehensive resolution needed. Hence, a pressing need exists for further exploration to grasp the genetic diversity and dynamics of *T. cruzi* during oral transmission events.

**Fig. 1:**
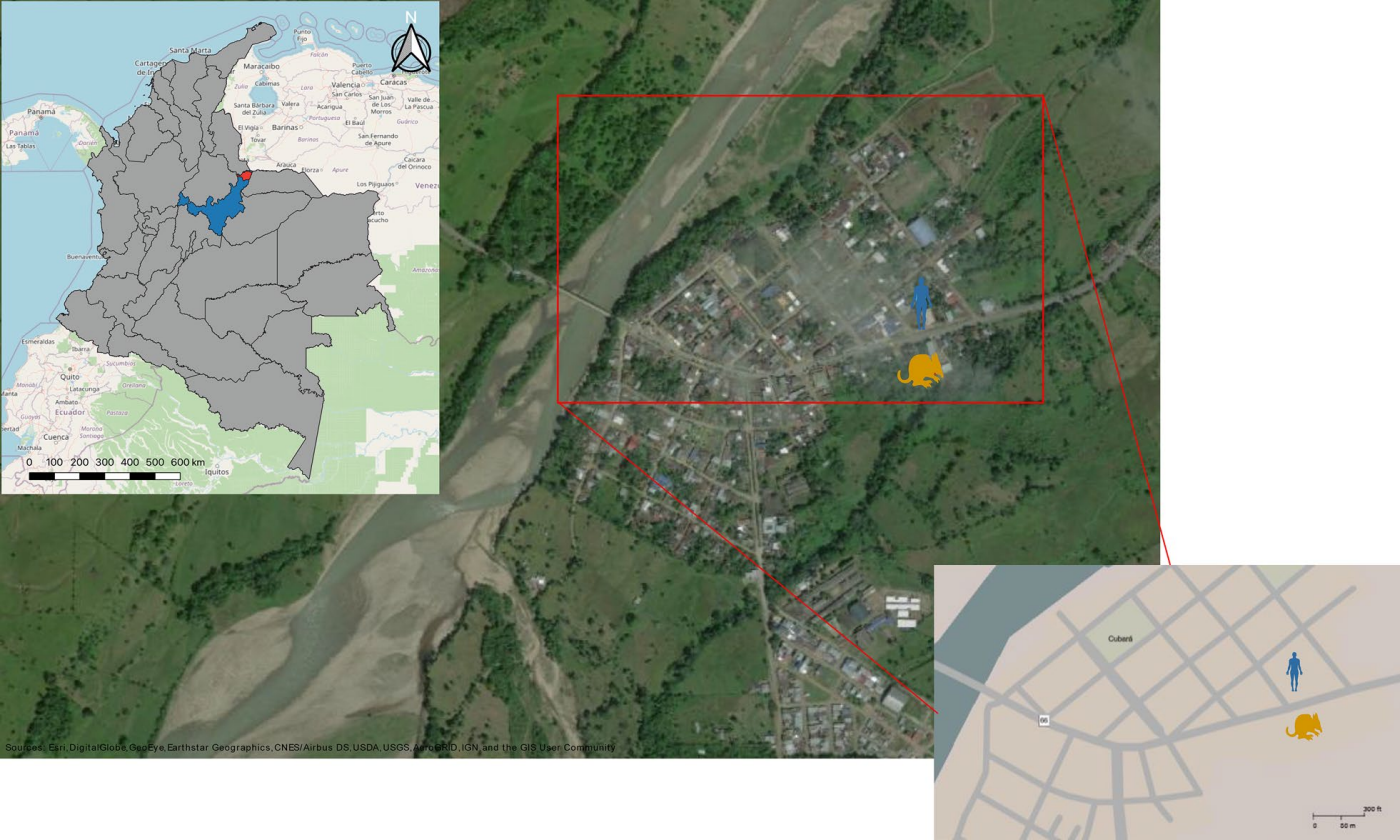
Map depicting the Chagas outbreak location in Cubará, Colombia. The map of Colombia highlights the Boyacá department in blue and the town of Cubará in red. The blue icon represents the index house of the patients on Cubará map, and the red icon depicts the location where marsupials were captured

In 2021, a Chagas disease outbreak occurred in Cubará, Colombia, detailed by Gutierrez et al. [15] (Fig. 1). Five family members exhibited Chagas-like symptoms, confirmed through microhematocrit, ELISA, CHAGATEK ELISA and qPCR, indicating *T. cruzi* infection. Investigation covered patient residences and surroundings, detecting *T. cruzi* in marsupials but not in a *Panstrongylus geniculatus* specimen collected in the zone. Conventional PCR identified mixed *T. cruzi* infections in patients and one marsupial specimen. However, due to the potential presence of diverse Trypanosomatid species and DTUs, amplicon-based sequencing (ABS) is crucial for gaining deeper insights, highlighting the importance of employing innovative strategies for complex scenarios.

ABS has already proven valuable in gauging the genetic diversity of Trypanosomatids like *Leishmania* and *T. cruzi*, yielding promising insights into transmission dynamics across humans, hosts, vectors and even maternal-fetal transmission cases [16–18]. ABS, targeting small regions like 18S ribosomal RNA and *TcSC5D* genes, showcased substantial genetic diversity in triatomines and congenital Chagas disease instances, hinting at specific genotypes linked to transmission [18, 19]. However, thus far, many studies have been confined to platforms such as Illumina, which might not be universally accessible and only facilitate short-read sequencing. Additionally, the application of long ABS to comprehensively assess all factors in oral outbreaks and the subsequent diversity loss over time, whether in infection or laboratory cultures, remains unexplored.

Since the COVID-19 pandemic, the Oxford nanopore (ON) sequencing platform has gained traction in regional health laboratories with limited access. This platform was incorporated thanks to funding projects targeting enhanced response to public health crises. Now, the ON technology can be extended to real-time monitoring of other endemic diseases, facilitating timely public health decisions. Thus, this research seeks to bridge a significant knowledge gap by leveraging the capabilities of long ABS to unveil a deeper comprehension of genetic diversity and transmission dynamics during *T. cruzi* oral transmission events.

Past studies focused on parasite presence, but our ABS-based investigation dives deeper, exploring *T. cruzi*’s genetic variations at a clone level during a recent acute Chagas disease outbreak. This approach illuminates genetic diversity implications for Chagas disease. We aim to guide effective strategies, deepen evolutionary insights and safeguard at-risk communities.

## Methods

### Chagas outbreak information and samples

A total of seven samples were gathered from human patients (*Homo sapiens*), alongside five samples from marsupials (*Didelphis marsupialis*). For detailed sample information, refer to Additional file 1: Table 1. Initial serum samples were obtained from two patients (C1 and C3) upon their arrival at the primary healthcare center. After hospitalization (2 days), serum samples were collected from all patients (C1post, C3post, C2, C6, C7). This longitudinal sampling approach provided insight into the progression of *T. cruzi* infection over time in patients. Marsupials’ blood samples, captured during the outbreak, were utilized as sources for DNA extraction (Z1–Z5) and hemoculture (Z1-post–Z3-post, Z5-post); the hemoculture was made in Tobbie/Liver infusion tryptose biphasic medium immediately after sample collection. Just four hemocultures were found positive, which offered an opportunity to study *T. cruzi* diversity effects under culture conditions. More information about sample collection's basic serological and molecular results can be found in Gutiérrez et al. [15].

### DNA extraction, long sequencing PCR and SL-IR sequencing

DNA extraction from serum of patients and marsupials employed the High Pure PCR Template Roche kit (Roche, Germany). The first part of the 18S rRNA gene (approximately 900 bp—205 informative SNPs) was amplified using the previously validated primers Tc18s_longFs_Fw (TCAGACGTAATCTGCCGCAA) and Tc18s_longFs_Rv (CCAACAAAAGCCGAAACGGT) from our Laboratory. The PCR mixture included LongAmp Taq 2X Master Mix. Thermal cycling conditions encompassed denaturation, annealing and extension steps. Amplified products were visualized on a 2% agarose gel. DNA controls from reference strains of *T. cruzi* DTUs were included for quality control, ensuring selective amplification and *T. cruzi* DNA presence verification. Additionally, a fragment of 300 to 350 base pairs was amplified using PCR techniques targeting the intergenic mini-exon gene region (SL-IR) with the primers TCC (5′-CCCCCCCTCCCAGGCCACACTG-3′), TC1M (5′-GTGTCCGCCACCTCCTTCGGGCC-3′) and TC2 (5′-CCTGCAGGCACACGTGTGTGTG-3′), following the protocol described by Souto [20]. The genotyping PCR products were subjected to electrophoresis, purified using ExoSAP-IT® and sequenced in both forward and reverse directions using the Sanger method by Macrogen (Seoul, South Korea).

### ON library preparation and sequencing

The PCR products were utilized to generate a sequencing library following the manufacturer’s instructions of the Ligation Sequencing Kit (SQK-LSK109, ONT, Oxford, UK). Initially, the PCR products underwent end-repair and A-tailing using the NEBNext End Repair/dA-tailing module. To incorporate barcodes, the end-prepped DNA was ligated using the NEBNext Ultra II Ligation Module Kit and PCR barcoding EXP-NBD196. Adapter ligation was performed subsequently using the NEBNext Quick Ligation Module Kit. The resulting library was loaded onto an R9.4 flow cell (FLO-MIN106) and sequenced on the MinIonMk1C instrument with MinKNOW software V22.10.7. Base-calling was conducted using Guppy v6.3.9, and reads below a minimum quality score of seven were excluded from further analysis.

### Reads bioinformatic analysis

Nanostat software assessed fastq file quality. Filtlong applied quality and size filters (mean score ≥ 10). Centrifuge software ensured accurate taxonomic assignment, emphasizing Trypanosomatids. Ugene aligned NCBI refseq 18S rRNA gene references via MAFFT (Additional file 1: Table 1). Centrifuge was running under parameters --min-totallen 100 --qc-filter and generated csv files filtered by hit length 70% and query length between 850 and 1200 bp (expected length of amplicon). Abundance visualization used R Studio’s ggplot package. In cases of unexpected assignments, they were confirmed via BLASTn of extracted reads including parameter -perc_identity 90 -evalue 0.05 -max_target_seqs 1. Consult our github to access the bioinformatic pipeline: https://github.com/gimur.

### Phylogenetic analysis

The reads obtained from the fastq files were sorted based on the results obtained from Centrifuge and BLASTn analyses of length (850 bp to 1200 bp), coverage (> 70%) and identity (> 90%) compared to the trypanosomatid data base. The reads were then assigned to the respective DTUs that were most commonly identified. The Ugene software platform was utilized, employing tools such as MAFFT for multiple sequence alignment and IQ-TREE for phylogenetic tree construction, incorporating the best model of substitution based on statistical tests obtained for each alignment. The resulting phylogenetic trees were further edited for visualization using the Itol software. A dissimilarity distance matrix was generated in Ugene through alignment and used to create a heatmap and dendrogram using average linkage clustering and Pearson correlation as the distance measure in Heatmapper [21]. Additionally, PCA was performed using R software (v. 4.2.2) with the “stats” package and “prcomp” function.

## Results

### 18S rRNA long amplicon-based sequencing reveals coinfection dynamics in patients and marsupials

After applying 18S rRNA long-amplicon-based sequencing and quality filtering, a significant number of reads per sample (ranging from 9325 to 29,823) passed the quality check. Centrifuge analysis focused on high-quality reads with expected lengths (around 900 bp) and optimal hit lengths (over 70%). To ensure accurate taxonomic assignments, a secondary BLASTn analysis against an in-house database was conducted for validation. All the detected DTUs and trypanosomatids per sample are summarized in Supplementary Table 3.

**Fig. 2:**
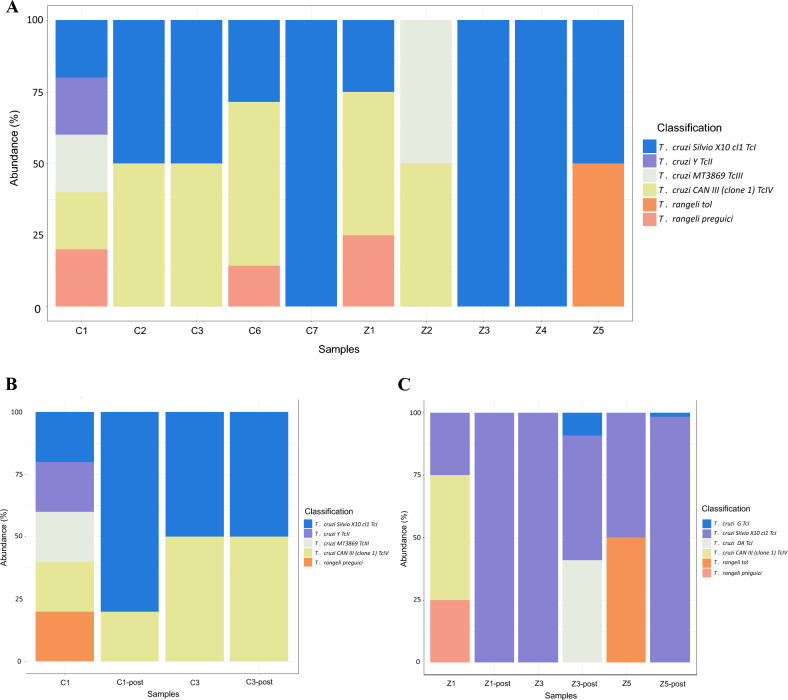
Trypanosomatids-ABS reveals high diversity of trypanosomatids and decrease in diversity during the Chagas oral outbreak in Cubará, Colombia. **A** Trypanosomatid diversity in blood samples from humans and marsupials. **B** Reduction in trypanosomatid diversity in human samples following infection (initial samples collected upon arrival at primary healthcare center and subsequently during hospitalization). **C** Loss of trypanosomatids in marsupial samples after culture. Initial serum samples for two patients (C1 and C3) upon their arrival at the primary healthcare center and after hospitalization from all patients (C1post, C3post, C2, C6, C7). Marsupial blood samples taken during the outbreak (Z1–Z5) and after hemoculture (Z1-post–Z3-post, Z5-post)

Strikingly, all patients were infected with the TcI DTU. Among them, four individuals had mixed infections with the TcIV DTU. Additionally, *T. rangeli* presence was detected in two patients. Similar results were observed by Gutierrez et al. [15] when employing conventional PCR genotyping on the same samples. Patient C1, immunosuppressed, displayed broader DTU infection, including TcII and TcIII (Fig. 2A). Among the marsupials, the TcI DTU was identified in four out of five samples, TcIV in two samples and TcIII in only one sample. Furthermore, *T. rangeli* was found in coinfection with *T. cruzi* in two samples, and a third coinfection event was observed involving two *T. cruzi* DTUs (TcIII and TcIV). Strikingly, marsupial Z1 displayed a similar pattern of infection involving DTUs TcI, TcIV and *T. rangeli*, mirroring the findings observed in two humans (Fig. 2A).

Sequential samples were collected from patients C1 and C3, initially after their first medical center visit and subsequently after 2 days of hospitalization (C1post and C3post). Notably, in immunosuppressed patient C1, a loss of diversity was observed in the second sample, with only the TcI and TcIV DTUs remaining (Fig. 2B). Regrettably, the loss of diversity could not be followed up until treatment and over time. This diversity loss was not observed in C3. In addition, blood samples from three captured marsupials were utilized to establish long-amplicon-based sequencing (long-ABS) and culture. Remarkably, the samples derived from culture exhibited a significant loss of diversity, with all three clones showing only the TcI DTU (Fig. 2C). This observation highlights the impact of culture on the diversity of *T. cruzi* populations.

### Comparative analysis of TcI DTU reveals commonalities between patients and marsupials

**Fig. 3:**
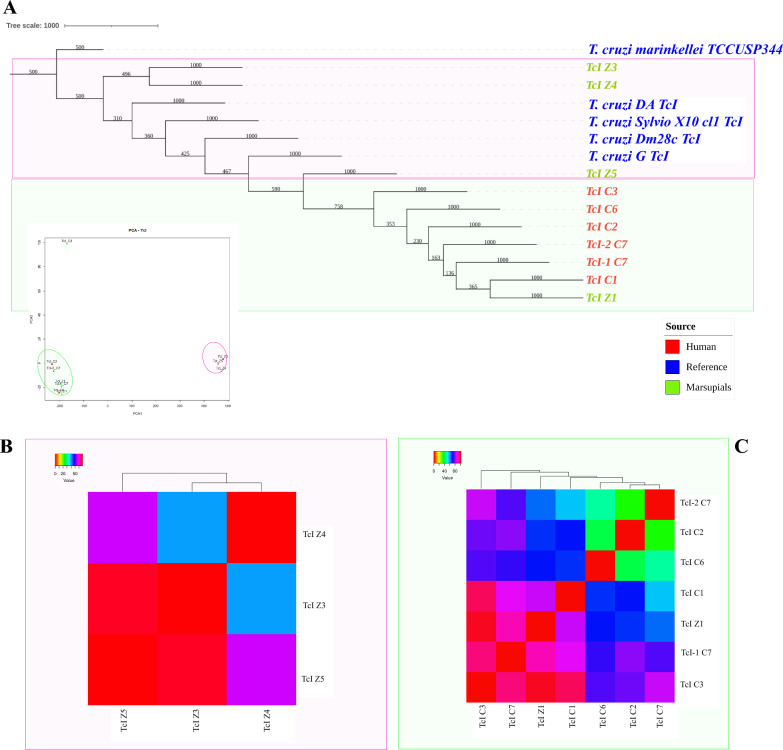
Comparative analysis of TcI DTU of *T. cruzi* reveals commonalities between patients and marsupials. **A** Phylogenetic analysis of TcI DTU of *Trypanosoma cruzi*. The reference sequences are in blue, sequences from human patients in red and sequences from marsupials in green. Cluster 1 is enclosed by a pink circle, while cluster 2 is represented by a green circle. **B** Matrix of dissimilarities based on Pearson correlations for cluster 1. **C** Matrix of dissimilarities based on Pearson correlations for cluster 2

Following the assignment and verification of reads, a phylogenetic analysis was conducted using 1000 bootstrap replicates. To confirm our findings, a reference sequence was included in the analysis. The reads assigned to the TcI DTU formed two distinct, well-defined clusters (Fig. 3A). Cluster 1 encompassed reads obtained from marsupials (Z3, Z4, Z5—depicted in pink), which grouped together with reference strains (blue color). Cluster 2 consisted of reads obtained from patients and marsupial Z1, which exhibited a strikingly similar pattern of coinfection and mixed infection to that observed in humans (depicted in green) (Fig. 3A). These results were corroborated using Sanger sequencing of the mini-exon gene (Fig. S1).

Our exploration extended further, diving into dissimilarity matrices within each cluster, followed by Pearson correlation analysis. In the first cluster, Z3 and Z4’s genetic scripts showcased closeness, differing by just 40 SNPs—as evidence of genetic connection. Z5 presented a unique pattern, weaving in 69 SNPs with Z3 and 56 SNPs with Z4, highlighting its distinct genetic signature (Fig. 3B). Within the same patient (TcI-1 C7, TcI-2 C7), 76 dissimilarities revealed genetic changes within a single host. Notably, TcI-2 C7 matched with C2 with only 32 dissimilarities, hinting at a unique genetic pattern. Genetic similarity was strongest among reads from C1, C2, C6 and TcI-2 C7, suggesting a potential shared source. Marsupial Z1’s genetic datat showed connections: 62 SNPs with TcI-2 C7, 66 with C2 and 70 with C6. However, TcI-1 C7 and C3 reads showed contrasting results in terms of dissimilarities (Fig. 3C).

### Phylogenetic analysis of DTU TcIV reveals associations between patients and marsupials

**Fig. 4:**
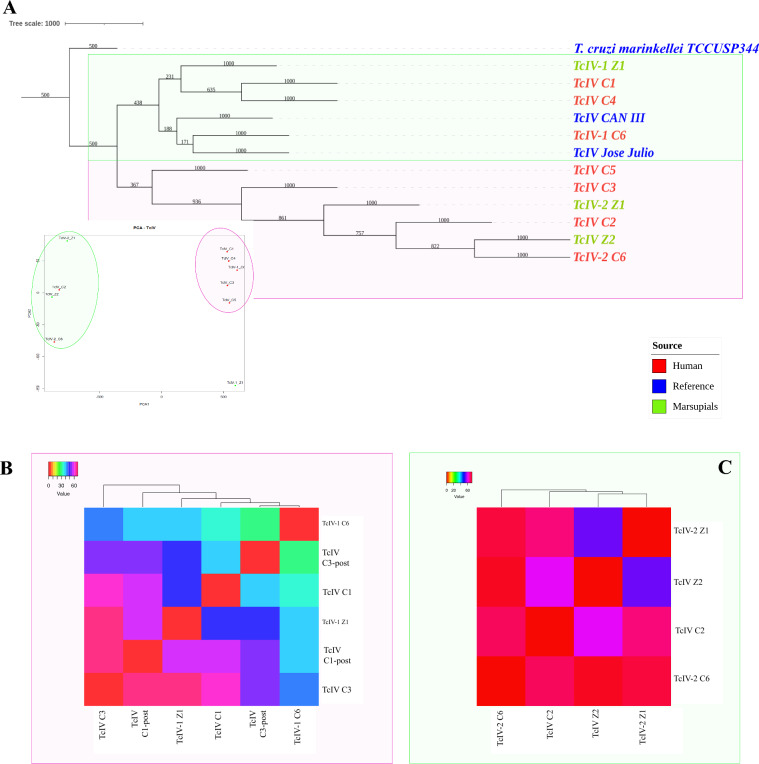
Phylogenetic analysis of DTU TcIV reveals associations between patients and marsupials. **A** Phylogenetic analysis of TcIV DTU of *Trypanosoma cruzi*. Reference sequence is in blue, sequences from human patients in red and sequences from marsupials in green. Cluster 1 is enclosed by a pink circle, while cluster 2 is represented by a green circle. **B** Matrix of dissimilarities based on Pearson correlations for cluster 1. **C** Matrix of dissimilarities based on Pearson correlations for cluster 2

In addition to the analysis of DTU TcI, a phylogenetic analysis was conducted for the reads assigned to DTU TcIV (Fig. 4A). Similar to the observations made for patient C7 within DTU TcI, we identified genetic differences in reads associated with patient C6 (TcIV-1 C6 and TcIV-2 C6) and marsupial Z1 (TcIV-1 Z1 and TcIV-2 Z1). The phylogenetic tree and PCA analysis exhibited two distinct clusters. In the first cluster (depicted in pink), the TcIV reference strains (shown in blue) grouped together with reads from the five samples, including C1, C3, TcIV-1 C6 and TcIV-1 Z1. Interestingly, within this cluster, the reads from the second samples of C1 and C3 (C1-post and C3-post) also clustered together. In the second cluster, the reads from human C2 and TcIV-2 C6 were closely related to reads from marsupial Z2 and TcIV-1 Z1. Notably, the reads from TcIV-2 C6 and Z1 formed a distinct branch within this cluster, suggesting a potential shared genetic lineage. These findings provide insights into the genetic relationships and associations between patients and marsupials within the DTU TcIV, highlighting the complexity of transmission dynamics and potential reservoir hosts in Chagas disease. These results were corroborated using Sanger sequencing of the mini-exon gene (Fig. S2).

Our genetic exploration revealed intriguing connections. In the first cluster, TcIV C3-post and TcIV-1 C6 differed by only 31 SNPs. Shared genetic links between C1 and TcIV-1 C6 (40 SNPs). Marsupial Z1 mirrored this pattern, differing by 39 SNPs from TcIV-1 C6, 48 SNPs from TcIV C3-post and 50 SNPs from C1. Between initial and subsequent samples from C1 and C3, we noted 55 and 56 SNPs of change, respectively. A notable 69-SNP divergence emerged between TcIV-1 Z1 and C3 within this cluster (Fig. 4B). In the second cluster, our dissimilarity analysis highlighted 54 to 70 SNPs between samples, irrespective of origin. In addition, between TcIV Z2 and TcIV-2 Z1 (54 SNPs). Likewise, TcIV Z2 and TcIV_C2 linked with 60 SNPs, unveiling shared genetic links (Fig. 4C).

### Exploring the diversity of *T. rangeli* in human and marsupials

**Fig. 5:**
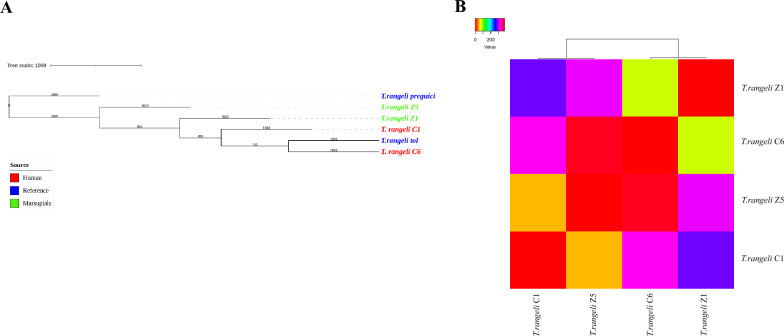
Exploring the diversity in *Trypanosoma rangeli* in humans and marsupials. **A** Phylogenetic analysis of *T. rangeli*. The reference sequence is in blue, sequences from human patients in red and sequences from marsupials in green. **B** Matrix of dissimilarities based on Pearson correlations for *T. rangeli*

To investigate the genetic diversity of *T. rangeli* in both human and marsupial hosts, we applied a similar approach of phylogenetic analysis and dissimilarity matrix. The phylogenetic tree revealed that the reference sequence of *T. rangeli*_Tol isolated from Colombia clustered together with samples obtained from human patients C1 and C6 as well as from marsupial Z1 (Fig. 5A). Additionally, the reads obtained from sample Z2 grouped with the other reference sequences indicating a shared genetic relationship (Fig. 5A). Consistent with the phylogenetic analysis, the dissimilarity matrix demonstrated the highest similarity between reads assigned to *T. rangeli* in the comparison between patient C1 and marsupial Z5, with only 45 SNPs of difference. This was followed by the comparison between patient C6 and marsupial Z1, exhibiting 81 SNPs of difference (Fig. 5B).

## Discussion

In this study, we utilized Long-18S rRNA Amplicon-Based Sequencing (ABS) to assess a wide range of samples with diverse origins and varying levels of parasite diversity and quantity. Our approach demonstrated remarkable sensitivity and precise resolution in analyzing the dynamics of *T. cruzi* transmission during the oral outbreak, encompassing samples from both humans and marsupials. This method yielded insights into the natural occurrence of coinfection and mixed infection of trypanosomatids in sylvatic hosts. Furthermore, it shed light on the subsequent reduction in diversity following human infection and culturing. These aspects may have been previously underestimated because of limitations in available genotyping methods, such as the lower accuracy and shorter read lengths of Oxford Nanopore sequencing as well as its inability to resolve mixed infections compared to the more precise PCR or MLST techniques in available genotyping methods.

The prevalence of DTU TcI in samples aligns with established data, reflecting its dominance in Colombia and its role in Chagas oral outbreaks [4, 22]. Notably, DTU TcIV and *T. rangeli* were found in patients and marsupials, indicating coinfection and mixed infection instances (Fig. 2). TcIV, less common but linked to oral Chagas, is noteworthy. Genetic similarities in trypanosomatids and DTUs between humans and marsupials are significant. *Trypanosoma rangeli* in both hosts complicates transmission and has been reported in few studies in human and skin of *Didelphis albiventris* [23, 24]. Phylogenetic analysis shows shared genetic lineage, while dissimilarity analysis implies genetic diversity within *T. rangeli*. Exploring interactions of *T. cruzi* and *T. rangeli* is key for Chagas disease insight [25]. Coinfection and mixed infection may impact the severity of clinical symptoms because of variations in parasite phenotypes, immune responses and tropism [26].

The detection of mixed infections involving different DTUs (TcI and TcIV) in patients and marsupials adds another layer of complexity to the transmission dynamics of *T. cruzi*. Four patients exhibited mixed infections, with the presence of both TcI and TcIV DTUs. Interestingly, marsupial Z1 displayed a similar pattern of mixed infection involving the same DTUs, reinforcing the potential role of marsupials in the transmission cycle. The dissimilarity analysis within clusters further supported the notion of shared infections, as the reads from different patients and marsupial Z1 exhibited low dissimilarities, indicating a potential shared source of infection. The strong pattern of DTUs and Trypanosomatid infection observed suggests a common origin of infection and potentially even cross-species transmission between the two groups. This interpretation gains further support from the genetic relationships unveiled by the phylogenetic analysis. The clustering of genetic sequences from both patients and marsupial Z1 strongly implies a genetic connection between these two entities (Figs. 3, 4). These findings point to the intriguing possibility of marsupials serving as reservoir hosts, actively contributing to the spread and transmission of *T. cruzi* during oral outbreak scenarios in the lack of evident vector transmission.

In fact, we made significant observations in the field. We identified a total of five qPCR *T. cruzi*-positive marsupials in the proximity of the patients’ residence, with four of them yielding positive results in subsequent hemoculture tests (Fig. 1; Additional file 1: Table 2). Interestingly, no *T. cruzi*-positive triatomine insects were discovered. This stark contrast underscores the substantial presence of sylvatic hosts in the region. Given that Cubará is a remote town with extensive sylvatic areas, this finding aligns with expectations. Notably, existing literature has provided support for and confirmation of the transmission of *T. cruzi* by the contamination of water and food with anal secretions, where infected forms of *T. cruzi* have been detected and observed, from infected opossums, and our results suggest the Cubará Chagas outbreak might have followed the same route [27, 28].

Intriguingly, the immunosuppressed patient C1 cast a spotlight on a broader spectrum of *T. cruzi* diversity, revealing the presence of not only TcI but also TcII, TcIII and even *T. rangeli*. This finding hints at the dynamic interplay between the host’s immune status and the diversity of *T. cruzi* infection. The impact of immune status on Chagas disease is a terrain ripe for exploration. Emerging evidence points to the heightened severity of Chagas disease in individuals coinfected with HIV, a combination that seems to exacerbate tissue invasion and lead to potentially lethal outcomes [29, 30]. Delving into the complex interplay between host immune responses and the development of mixed infections could shed light on the factors that drive disease progression. This also might imply that a diverse initial pathogen load is subjected to selective filtration within the infected host, ultimately leading to a constrained infection outcome.

In human samples, we have observed a loss of DTU TcIV and *T. rangeli* after 2 days of infection, while DTU TcI remains dominant (Fig. 2B). This finding is highly intriguing, particularly considering the prevalence of TcI in Colombia. The underlying mechanism by which the immune system or other factors might lead to a bottleneck effect on *T. cruzi* DTU diversity in humans remains uncertain. To our knowledge, this marks the first report of diversity loss in human blood samples, potentially shedding light on the predominance of TcI in reports of Chagas acute outbreaks. Regarding the presence of *T. cruzi* DTUs II and III in the immunosuppressed host, it is noteworthy that these DTUs, while less common, are the second most reported in Colombia after TcI [22]. Their detection, despite their lower prevalence, could help explain the observed loss of diversity in the patient. This finding suggests that DTUs II and III, although present at lower frequencies, might play a role in the diversity loss observed in this patient. This case highlights the need for further investigation to better understand the full range of *T. cruzi* DTUs present and their distribution within the area, particularly in relation to immunological factors and tissue tropism.

One plausible hypothesis is the swift migration of DTU TcIV to specific tissues. In fact, studies involving oral infection of mice with TcIV oral outbreak strains have shown a notable parasite abundance in tissues post-infection [31]. If this scenario holds true, it prompts us to question whether we have underestimated Chagas diversity by solely relying on blood samples. The histotrophic of distinct *T. cruzi* DTUs in humans has indeed been observed [10]. However, addressing this question is challenging because of the complexity of obtaining cardiac and gastrointestinal tissues from patients given the ethical considerations involved. These findings pose intriguing inquiries and emphasize the need for continued exploration into the interplay among various factors influencing DTU infection dynamics and diversity in Chagas disease, particularly during oral outbreaks. Gaining a better understanding of the mechanisms underlying diversity loss in blood and how different DTUs behave in diverse tissues could hold significant implications for disease diagnosis, treatment and control.

The reduction in diversity we observed in the cultured samples from marsupials sheds light on the significant impact that laboratory techniques can have on the diversity of *T. cruzi* populations [32]. This phenomenon has been recognized and discussed in previous studies highlighting the potential for culture methods to favor the growth of certain DTUs, which might then skew our view of the entire parasite population. This insight is a reminder of the potential limitations posed by culture-based approaches when investigating *T. cruzi* diversity and transmission dynamics. To gain a more holistic understanding of the parasite population, it is crucial to be mindful of these biases introduced by culture techniques. Innovative long-amplicon-based sequencing methods provide a more comprehensive understanding of *T. cruzi* diversity and transmission dynamics. These advanced techniques help reveal the complexity of the parasite’s genetic landscape.

In our study, we used Oxford Nanopore sequencing, which is advantageous because of its longer read lengths and real-time data acquisition, allowing for exploration of complex genetic landscapes. However, it has drawbacks compared to Illumina sequencing, including higher error rates and lower accuracy in base calling, which can impact the reliability of detecting genetic variations. Conversely, Illumina sequencing is known for its high accuracy and comprehensive coverage but faces limitations such as shorter read lengths and challenges in detecting structural variations, which are crucial for distinguishing between *T. cruzi* DTUs. Our focus on Oxford Nanopore sequencing and only two molecular markers is a limitation of our study, offering a narrower perspective compared to more extensive genomic analyses. Additionally, using serum instead of whole blood for PCR analysis is another limitation, as serum is less effective for detecting *T. cruzi* because of its lower sensitivity in PCR-based diagnostics. This choice may have contributed to the observed loss of diversity in our results. Future research should combine different sequencing technologies and employ broader genomic analyses to achieve a more detailed understanding of *T. cruzi* and similar pathogens, ultimately enhancing our knowledge and informing more effective control strategies.

## Conclusions

The findings of this study, based on Trypanosomatids ABS-long, shed light on the intricate dynamics of *T. cruzi* during acute outbreaks and provide valuable insights for control and prevention. This suggests the implementation of this technique to evaluate future Chagas outbreaks. The identification of marsupials as potential reservoir hosts emphasizes the importance of considering alternative transmission routes beyond triatomine vectors. Efforts should be directed toward understanding the interactions between different DTUs and other trypanosome species as well as the impact of host immune response, tissue tropism and laboratory techniques on the diversity of *T. cruzi* populations. Future studies should also explore the potential involvement of other wildlife species in the transmission cycle and investigate the genetic diversity and virulence factors of *T. cruzi* and *T. rangeli* strains to enhance our understanding of Chagas disease epidemiology and develop effective control measures.

## Supplementary Information

Additional file 1: Table S1. Trypanosomatid sequences included as reference during the bioinformatic analysis.

Additional file 2: Table S2. Sample information from human and marsupial samples collected.

Additional file 3: Supplementary Table 3. DTUs and trypanosomatids detected in humans and marsupials.

Additional file 4: Supplementary Figure 1. Phylogenetic tree comparison between 18S RNA Oxford Nanopore and mini-exon Sanger sequencing for TcI sequences. A. 18S nanopore reads. B. Mini-exon Sanger sequencing.

Additional file 5: Supplementary Figure 2. Phylogenetic tree comparison between 18S RNA Oxford Nanopore and Mini-exon Sanger sequencing for TcIV sequences. A. 18S nanopore reads. B. Mini-exon Sanger sequencing.

## Data Availability

All the data generated in this project were uploaded under the Bioproject PRJEB70421. No datasets were generated or analyzed during the current study.

## Abbreviations

DTU: Discrete typing unit
ABS: Amplicon-based sequencing
ON: Oxford nanopore
C: Patients
Z: Marsupials
PCA: Principal component analysis
