# Supplementary material for: Exploring Trypanosoma cruzi transmission dynamics in an acute Chagas disease outbreak using next-generation sequencing
Source: Parasit Vectors. 2024 Sep 18;17:395. doi: 10.1186/s13071-024-06445-9 (PMC11409604; doi:10.1186/s13071-024-06445-9)
Supplement: Supplementary file 1 — Additional file 1: Table S1. Trypanosomatid sequences included as reference during the bioinformatic analysis. [file 13071_2024_6445_MOESM1_ESM.docx]

**Supplementary table 1.** Trypanosomatids sequences included as reference during the bioinformatic analysis.

| **GenBank code** | ***Sequence name*** |
| --- | --- |
| AJ012411.1 | *Trypanosoma conorhini_USP* |
| AJ009148.1 | *Trypanosoma cruzi _CAN_III_(clone_1)_TcIV* |
| AF245382.1 | *Trypanosoma cruzi _Dm28c_TcI* |
| AY785564.1 | *Trypanosoma cruzi _Esmeraldo_cl3_TcII* |
| AF239981.1 | *Trypanosoma cruzi _G_TcI* |
| FJ001664.2 | *Trypanosoma cruzi _marinkellei_TCCUSP344* |
| AF288660.1 | *Trypanosoma cruzi _MT3663_TcIII* |
| AF303660.1 | *Trypanosoma cruzi _MT3869_TcIII* |
| AY785578.1 | *Trypanosoma cruzi _strain_M6241_cl6_TcIII* |
| AF301912.1 | *Trypanosoma cruzi _Y_TcII* |
| AF303659.1 | *Trypanosoma cruzi Silvio_X10_cl1_TcI* |
| *Inhouse* | *Trypanosoma cruzi TcBat_CHAQ12* |
| *Inhouse* | *Trypanosoma cruzi TcVI Tulahuen* |
| LC326397.1 | *Trypanosoma dionisii_MPMJPN21385* |
| AJ009152.1 | *Trypanosoma dionisii_PJ* |
| FJ001666.2 | *Trypanosoma dionisiiTCCUSP211* |
| JN040987.1 | *Trypanosoma erneyi_TCC1293* |
| JN040988.1 | *Trypanosoma erneyi_TCC1294* |
| AY491767.1 | *Trypanosoma rangeli_preguici* |
| *Inhouse* | *Trypanosoma rangeli_Tol* |
| AJ009166.1 | *Trypanosoma vespertilionis_P14* |
| KM391827.1 | *Trypanosoma vivax_BOV/ET/2012/AAU-CVMA/004_clone_2* |
| KM391829.1 | *Trypanosoma vivax_isolation Y486(ILRAD700)_clone_2* |
| AJ009142.1 | *Trypanosoma brucei_rhodesiense_UTRO_2509* |
| KX007997.1 | *Trypanosoma brucei_rhodesiense* |
| MN446740.1 | *Trypanosoma evansi_haB* |
| KY114578.1 | *Trypanosoma evansi_PKG* |
| AJ009163.1 | *Trypanosoma theileri_TREU_124* |
| KR024688.1 | *Trypanosoma theileri_G24* |
| GQ920678.1 | *Leishmania_aethiopica* |
| GQ332354.1 | *Leishmania_amazonensis* |
| GQ332355.1 | *Leishmania_braziliensis* |
| GQ332357.1 | *Leishmania_chagasi* |
| GQ332356.1 | *Leishmania_donovani* |
| GQ332358.1 | *Leishmania_guyanensis* |
| GQ332359.1 | *Leishmania_infantum* |
| GQ332361.1 | *Leishmania_major* |
| GQ332360.1 | *Leishmania_mexicana* |
| GQ332362.1 | *Leishmania_panamensis* |
| JN003595.1 | *Leishmania_panamensis* |
| GQ332363.1 | *Leishmania_tropica* |
