# Supplementary material for: Exploring Trypanosoma cruzi transmission dynamics in an acute Chagas disease outbreak using next-generation sequencing
Source: Parasit Vectors. 2024 Sep 18;17:395. doi: 10.1186/s13071-024-06445-9 (PMC11409604; doi:10.1186/s13071-024-06445-9)
Supplement: Supplementary file 2 — Additional file 2: Table S2. Sample information from human and marsupial samples collected. [file 13071_2024_6445_MOESM2_ESM.docx]

**Supplementary table 2.** Sample information from humans and marsupials’ samples collected.

| **Sample** | **Host** | **Origen place** | **Collection place** | **Acronym** | **Microhematocrit** | **qPCR** | **ELISA** | **Hemoculture** |
| --- | --- | --- | --- | --- | --- | --- | --- | --- |
| 1 | *Homo sapiens* | Town | First healthcare center | C1 | + | + | - | NR |
| 2 | *Homo sapiens* | Town | Hospitalization | C2 | + | + | - | NR |
| 3 | *Homo sapiens* | Town | First healthcare center | C3 | + | + | + | NR |
| 4 | *Homo sapiens* | Town | Hospitalization | C3-post  (10 days post hospitalization) | NR | + | + | NR |
| 5 | *Homo sapiens* | Town | Hospitalization | C1-post  (10 days post hospitalization) | NR | + | - | NR |
| 6 | *Homo sapiens* | Town | Hospitalization | C6 | + | + | - | NR |
| 7 | *Homo sapiens* | Town | Hospitalization | C7 | + | + | + | NR |
| 8 | *Didelphis marsupialis* | Town | Town | Z1 | NR | + | NR | + |
| 9 | *Didelphis marsupialis* | Town | Town | Z2 | NR | + | NR | + |
| 10 | *Didelphis marsupialis* | Town | Town | Z3 | NR | + | NR | + |
| 11 | *Didelphis marsupialis* | Town | Town | Z4 | NR | + | NR | - |
| 12 | *Didelphis marsupialis* | Town | Town | Z5 | NR | + | NR | + |
| 13 | *Panstrongylus geniculatus* | Town | Town | - | NR | - | NR | NR |

*NR: Non-realized
