# Supplementary material for: Exploring Trypanosoma cruzi transmission dynamics in an acute Chagas disease outbreak using next-generation sequencing
Source: Parasit Vectors. 2024 Sep 18;17:395. doi: 10.1186/s13071-024-06445-9 (PMC11409604; doi:10.1186/s13071-024-06445-9)
Supplement: Supplementary file 3 — Additional file 3: Supplementary Table 3. DTUs and trypanosomatids detected in humans and marsupials. [file 13071_2024_6445_MOESM3_ESM.docx]

**Supplementary Table 3.** DTUs and trypanosomatids detected in human and marsupial.

| Sample | *T.cruzi_can_III_(clone_1)_TcIV* | *T.cruzi_DA_TcI* | *T.cruzi_G_TcI* | *T.cruzi_MT3869_TcIII* | *T.cruzi_Y_*  *TcII* | *T.cruzi Silvio_X10_cl1_TcI* | *T.rangeli_*  *preguici* | *T.rangeli_*  *tol* |
| --- | --- | --- | --- | --- | --- | --- | --- | --- |
| C1 | x |  |  | x | x | x | x |  |
| C1-post | x |  |  |  |  | x |  |  |
| C2 |  |  |  |  |  | x |  |  |
| C3 | x |  |  |  |  | x |  |  |
| C3-post | x |  |  |  |  | x |  |  |
| C6 | x |  |  |  |  | x | x |  |
| C7 |  |  |  |  |  | x |  |  |
| Z1 | x |  |  |  |  | x | x |  |
| Z1-Post |  |  |  |  |  | x |  |  |
| Z2 | x |  |  | x |  |  |  |  |
| Z3 |  |  |  |  |  | x |  |  |
| Z3-post |  | x | x |  |  | x |  |  |
| Z4 |  |  |  |  |  | x |  |  |
| Z5 |  |  |  |  |  | x |  | x |
| Z5-post |  | x |  |  |  | x |  |  |
