# Supplementary material for: Exploring Trypanosoma cruzi transmission dynamics in an acute Chagas disease outbreak using next-generation sequencing
Source: Parasit Vectors. 2024 Sep 18;17:395. doi: 10.1186/s13071-024-06445-9 (PMC11409604; doi:10.1186/s13071-024-06445-9)

**Supplementary Figure 1.** Phylogenetic three **c**omparison between 18S RNA oxford nanopore and miniexon Sanger sequencing for TcI sequences. A. 18S nanopore reads. B. Miniexon Sanger sequencing.


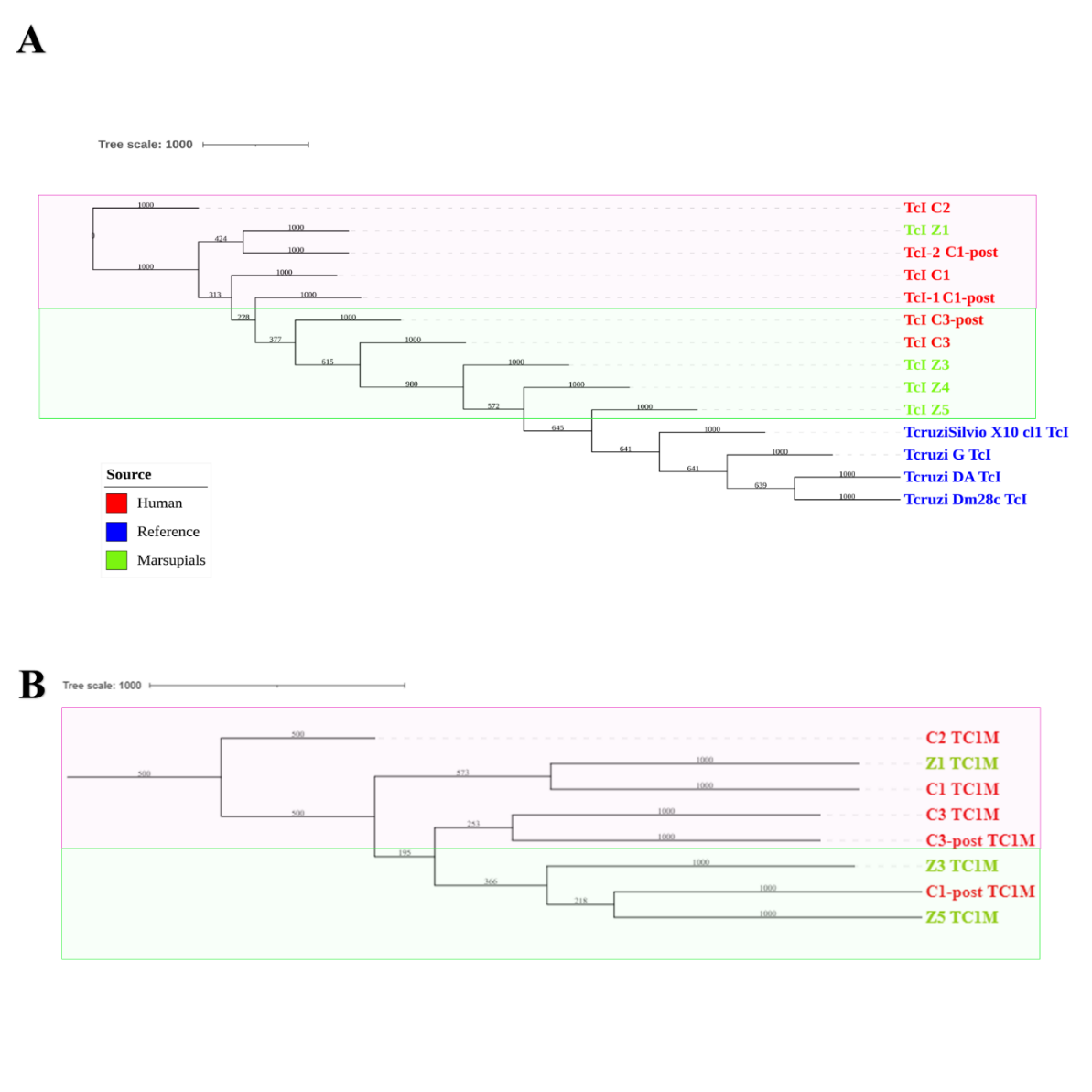

Supplement: Supplementary file 4 — Additional file 4: Supplementary Figure 1. Phylogenetic tree comparison between 18S RNA Oxford Nanopore and mini-exon Sanger sequencing for TcI sequences. A. 18S nanopore reads. B. Mini-exon Sanger sequencing. [file 13071_2024_6445_MOESM4_ESM.docx]
